# The use of low level laser therapy in the treatment of temporomandibular joint disorders. Review of the literature

**DOI:** 10.4317/medoral.18794

**Published:** 2013-05-31

**Authors:** Judit Herranz-Aparicio, Eduardo Vázquez-Delgado, Josep Arnabat-Domínguez, Antoni España-Tost, Cosme Gay-Escoda

**Affiliations:** 1 DDS. Fellow of the Master degree program of Oral Surgery and Implantology, School of Dentistry, University of Barcelona; 2 DDS.MS. Head of the TMJ and Orofacial Pain Unit of the Master of Oral Surgery and Implantology. University of Barcelona, School of Dentistry. Investigator of the IDIBELL Institute. Specialist of the Orofacial Pain Unit of the Teknon Medical Center. Barcelona; 3 DDS. MD, MS. PhD. Associate Professor of Oral Surgery. Co-director of the Master in Lasers in Dentistry. Barcelona University, School of Dentistry. Investigator of the IDIBELL Institute; 4 DDS. MD, MS. PhD. Associate Professor of Oral Surgery. Professor of the Master of Oral Surgery and Implantology. Director of the Master in Lasers in Dentistry. Coordinator of the European Master’s Degree in Oral Laser Applications (EMDOLA). Barcelona University, School of Dentistry. Investigator of the IDIBELL Institute; 5 MD, DDS, MS. PhD. Chairman and Professor of Oral and Maxillofacial Surgery. Director of the Master of Oral Surgery and Implantology. Barcelona University, School of Dentistry. Coordinating/ Investigator of the IDIBELL Institute. Head of the Department of Oral and Maxillofacial Surgery and Coordinator of the Orofacial Pain Unit, Teknon Medical Center. Barcelona, Spain

## Abstract

Introduction: The temporomandibular disorders (TMDs) have been identified as the most important cause of pain in the facial region. The low level laser therapy (LLLT) has demonstrated to have an analgesic, anti-inflammatory and biostimulating effects. The LLLT is a noninvasive, quick and safe, non-pharmaceutical intervention that may be beneficial for patients with TMDs. However the clinical efficiency of LLLT in the treatment of this kind of disorders is controversial. Objectives: Literature review in reference to the use of LLLT in the treatment of TMDs, considering the scientific evidence level of the published studies. Material and Methods: A MEDLINE and COCHRANE database search was made for articles. The keywords used were “temporomandibular disorders” and “low level laser therapy” or “phototherapy” and by means of the Boolean operator “AND”. The search provided a bank of 35 articles, and 16 relevant articles were selected to this review. These articles were critically analyzed and classified according to their level of scientific evidence. This analysis produced 3 literature review articles and 13 are clinical trials. The SORT criteria (Strength of Recommendation Taxonomy) was used to classify the articles. Results: Only one article presented an evidence level 1, twelve presented an evidence level 2, and three presented an evidence level 3. According to the principle of evidence-based dentistry, currently there is a scientific evidence level B in favor of using LLLT for treatment of TMDs. Discussion and conclusions: Publications on the use of LLLT for treatment of TMDs are limited making difficult to compare the different studies due to the great variability of the studied variables and the selected laser parameters. The great majority of the studies concluded that the results should be taken with caution due to the methodological limitations.

** Key words:**Low level laser therapy; phototherapy; temporomandibular joint disorders.

## Introduction

Temporomandibular disorders (TMDs) is a collective term that includes disorders of the temporomandibular joint (TMJ), and of the masticatory muscles and their associated structures; characterized by pain, joint sounds, and restricted mandibular movement (1,2). TMD etiology is currently known to be multifactorial, including the presence of parafunctional habits, trauma stress, as well as emotional, systemic, hereditary, and occlusal factors (2).The etiology is related to an association of predisposing factors that increase the risk of TMD, initiating factors that cause the onset of TMD, and perpetuating factors that interference with healing or enhance TMD progression (3). Epidemiological studies show that about 75% of the population presents one sign of TMD and 35 % present at least one symptom, however, only a minor percentage of the population, 3-7%, presents problems severe enough to look for treatment for TMD (4,5).

There is still a lack of consensus on the classification of TMD, largely because there is unclear etiology and clinical findings can result from different causes, including psychological causes. One commonly used diagnostic scheme intended for research purposes is the Research Diagnostic Criteria for TMD (RDC/TMD) (6). This standardizes the clinical examination of patients with TMD, improves reproducibility among clinicians, and facilitates comparison of results among researchers (7).

Aggressive and irreversible treatments, such as complex occlusal therapies and surgeries should be avoided. Nonsurgical treatment of TMDs generally consists of medication, such as nonsteroidal anti-inflammatory drugs (NSAIDs) and antidepressants, splint therapy or/and physiotherapy. NSAIDs may reduce the inflammation but may also increase the risk of complications, such as gastric ulcer and nephrotoxicity. Other treatments used are physical therapy (electrotherapy, ultrasound, acupuncture and laser), treatment of parafunctional activities and alternatives therapies. Physical therapy is used in the treatment of TMD because of its analgesic, myorelaxing, anti-inflammatory and stimulations effects. Low level laser therapy (LLLT) is an option for the treatment of musculoskeletal disorders, it is easy application, limited treatment time and minimum contraindications, due to its analgesic, anti-inflammatory and regenerative effects (3,4,8).

The clinical efficacy of LLLT for the treatment of TMDs is controversial. Some authors reported best results comparing the LLLT with a placebo control group, while others found no significant differences.

According to some authors there is considerable diversity in the results reported, depending on parameters and methodology used.

The aim of our study is to make a review of the literature published on the use of LLLT for the treatment of TMDs, considering the level of scientific evidence according to the principals of evidence-based dentistry.

## Material and Methods

A MEDLINE search was made for articles without restriction in year publication. The keywords used were “temporomandibular disorders” and “low level laser therapy” or “phototherapy” and by means of the Boolean operator “AND”. The literature identified was then limited to studies in humans and articles written in English. The same process was used in the COCHRANE database of the Cochrane Oral Health Group. Two authors analyzed the abstracts to verify that the articles obtained were pertinent to the topic under study. The irrelevant articles were discarded. Next, the same two authors independently stratified the scientific articles according to their level of scientific evidence using the SORT criteria (Strength of Recommendation Taxonomy). Subsequently the authors compared their results; in the event of disagreement the results were discussed. If no consensus regarding the level of scientific evidence of a certain article was possible, a third author was included in the discussion. Subsequently, a recommendation was given for or against the use of LLLT in the treatment of TMD according to the level of scientific evidence of the articles analyzed.

## Results

The MEDLINE search for TMDs and LLLT or photherapy when were cross provided a bank of 35 articles. Next, the abstracts of each article were analyzed to determine if they were pertinent to the topic under study. The search in the COCHRANE database provided no relevant articles that agreed with the search criteria of this study. After this process 16 relevant articles remained. These articles were critically analyzed and classified according to their level of scientific evidence. This analysis produced 3 literature review articles and 13 are clinical trials.

Description of studies.

1. Bjordal JM, Couppé C, Chow RT, Tunér J, Ljunggren EA. Literature systematic review. Evidence level 2.

2. Medlicott MS, Harris SR. Literature systematic review. Evidence level 2.

3. McNeely ML, Armijo Olivo S, Magee DJ. Literature systematic review. Evidence level 2.

4. De Medeiros JS, Vieira GF, Nishimura PY. Clinical trial. Evidence level 3.

5. Carvalho CM, de Lacerda JA, dos Santos Neto FP, Cangussu MC, Marques AM, Pinheiro AL. Clinical trial. Evidence level 3.

6. Fikácková H, Dostálová T, Navrátil L, Klaschka J. Clinical trial. Evidence level 2.

7. Çetiner S, Kahraman SA, Yücetaş S. Evidence level 2. Clinical trial. Evidence level 2.

8. Núñez SC, Garcez AS, Suzuki SS, Ribeiro MS. Clinical trial. Evidence level 3.

9. Venancio Rde A, Camparis CM, Lizarelli RF. Clinical trial. Evidence level 1.

10. Emshoff R, Bösch R, Pümpel E, Schöning H, Strobl H. Clinical trial. Evidence level 2.

11. Kato MT, Kogawa EM, Santos CN, Conti PCR. Clinical trial. Evidence level 2.

12. Hotta PT, Hotta TH, Bataglion C, Bataglion SA, Coronatto EAS, Siesseré S, Regalo SCH. Clinical trial. Evidence level 2.

13. Katsoulis J, Ausfeld- Hafter B, Windecker-Gétz I, Katsoulis K, Blagojevic N, Mericske-Stern R. clinical trial. Evidence level 2.

14. Mazzetto MO, Hotta TH, Pizzo RCA. Clinical trial. Evidence level 2.

15. Shirani AM, Gutknecht N, Taghizadeh M, Mir M. Clinical trial. Evidence level 2.

16. Kulekcioglu S, Sivrioglu K, Ozcan O, Parlak M. Clinical trial. Evidence level 2.

The results of the clinical trials that study the effects of LLLT are summarized in  Table 1 and  Table 1 continued, the results of studies that compare LLLT with the use of TENS application are summarized in  Table 2, the results of studies that compare LLLT with the use of laser acupuncture are summarized  Table 3 and the results of laser application parameters are summarized in  Table 4. In accordance with the principals of evidence- based dentistry, the analysis produced a level B recommendation strength in favor of using LLLT in the treatment of TMDs. However, these results should be taken with caution since these recommendations are based on studies with important methodological defects such as insufficient sample size and/or lack of homogeneity among the studied populations or the laser application parameters.

**Table 1:**
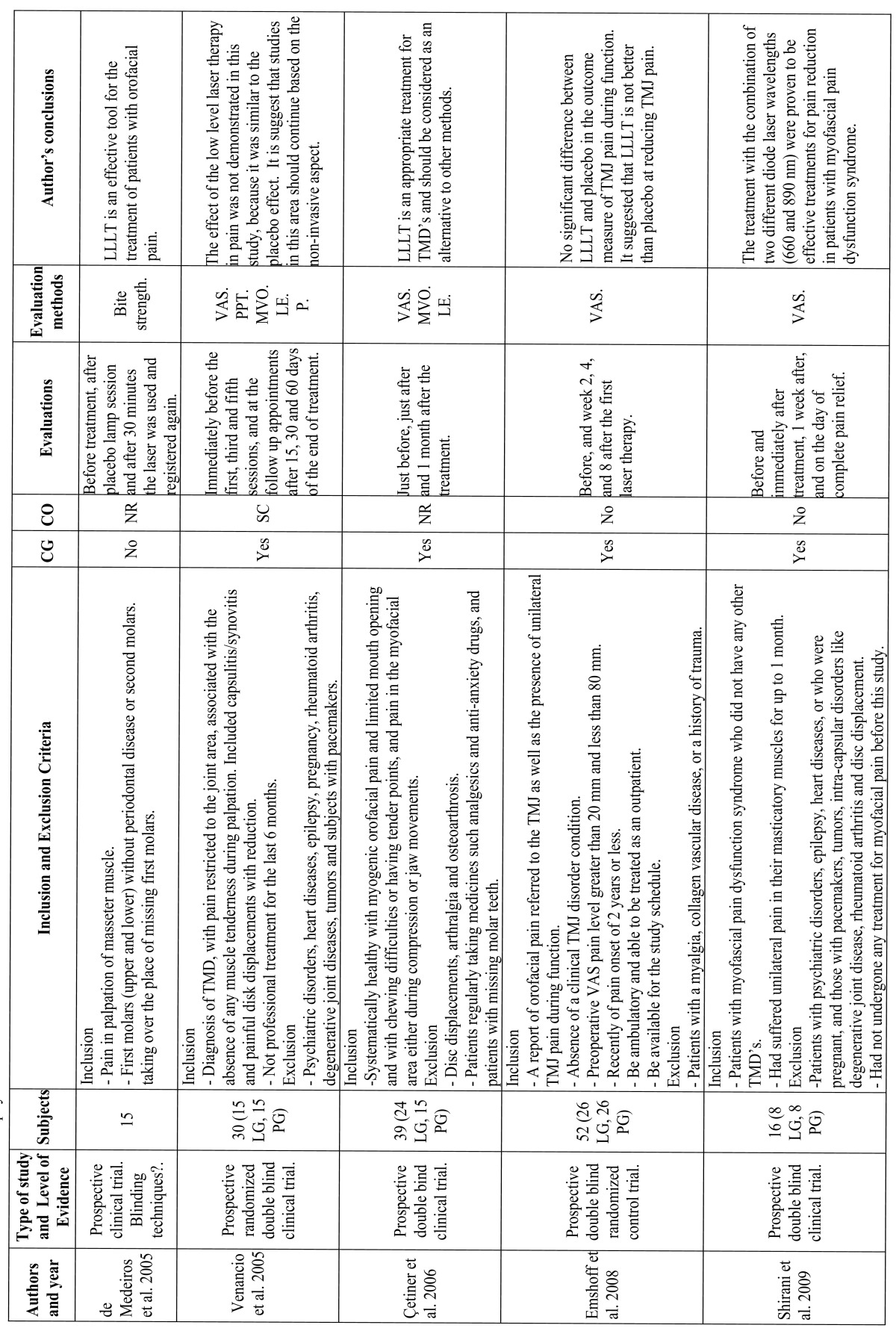
Low level laser theraphy clinical trials.

**Table 1 continued:**
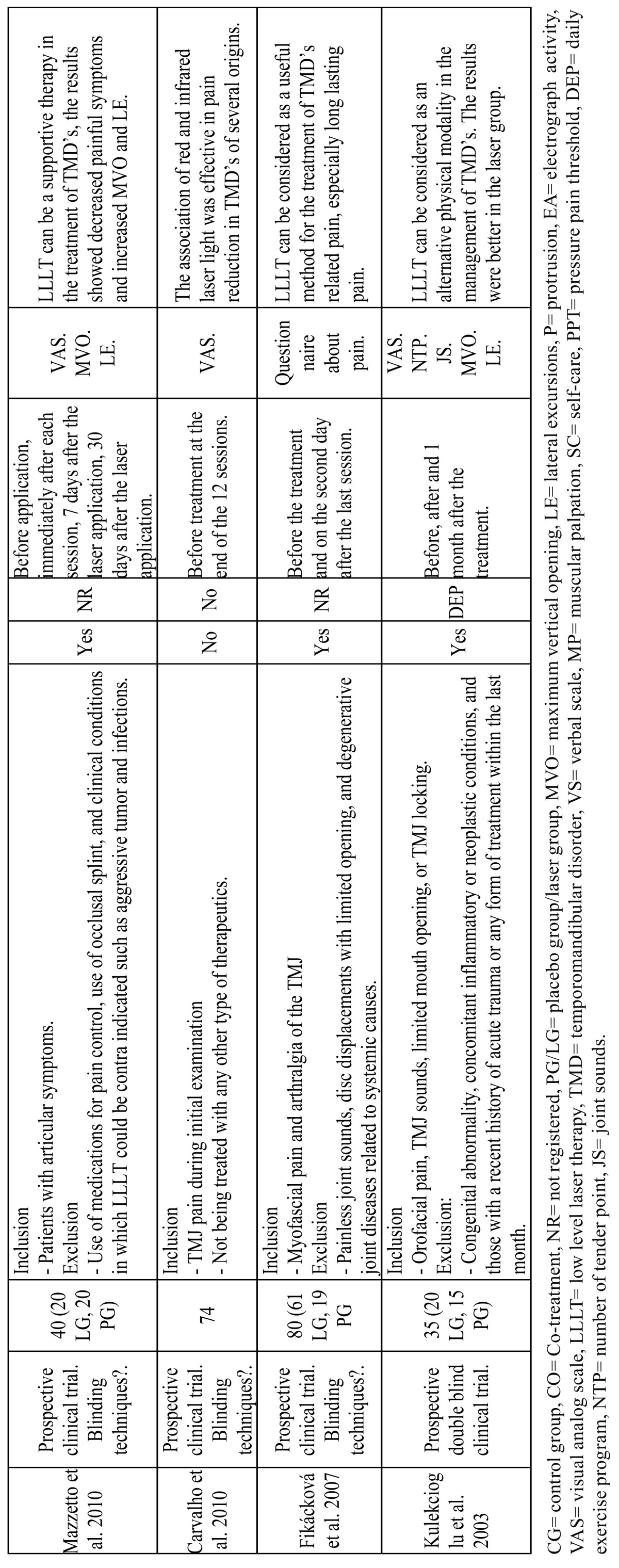
Low level laser theraphy clinical trials.

**Table 2:**
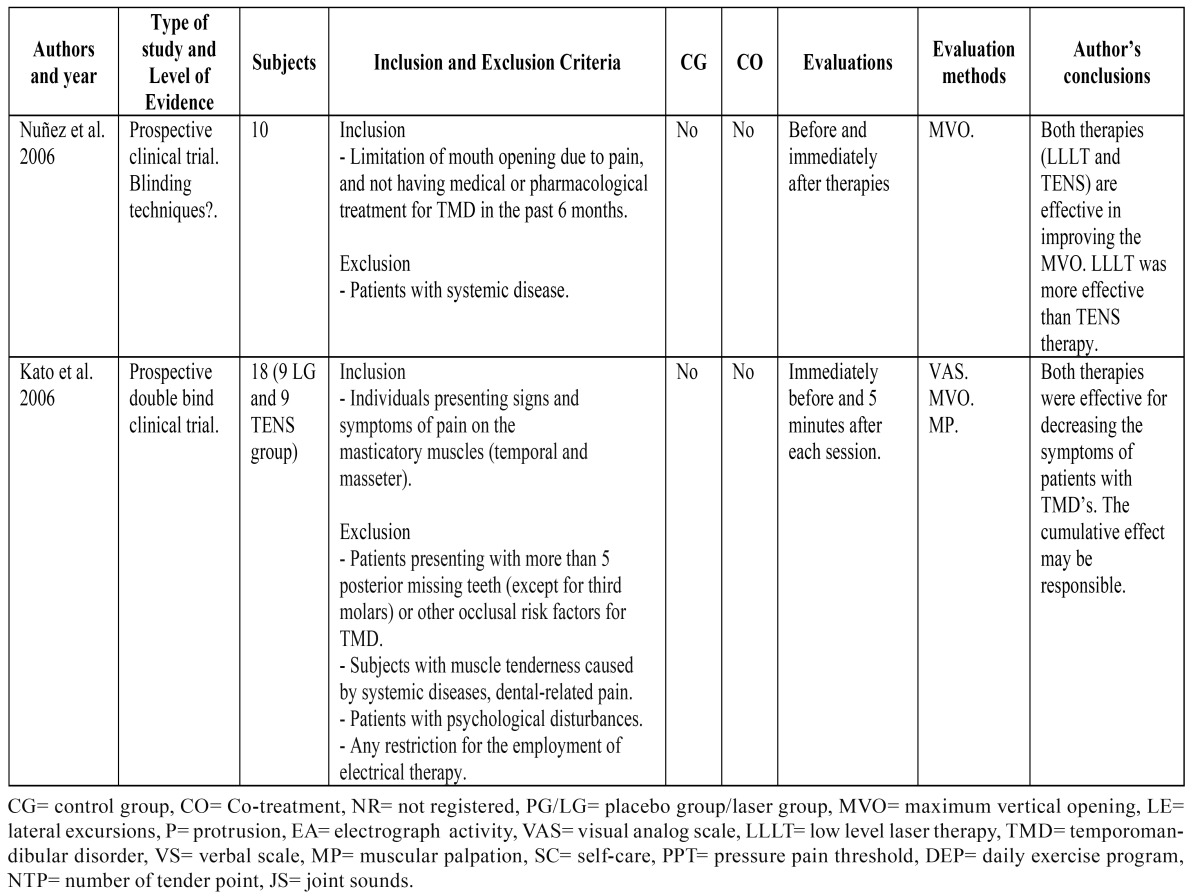
Clinical trials with TENS and low level laser application.

**Table 3:**
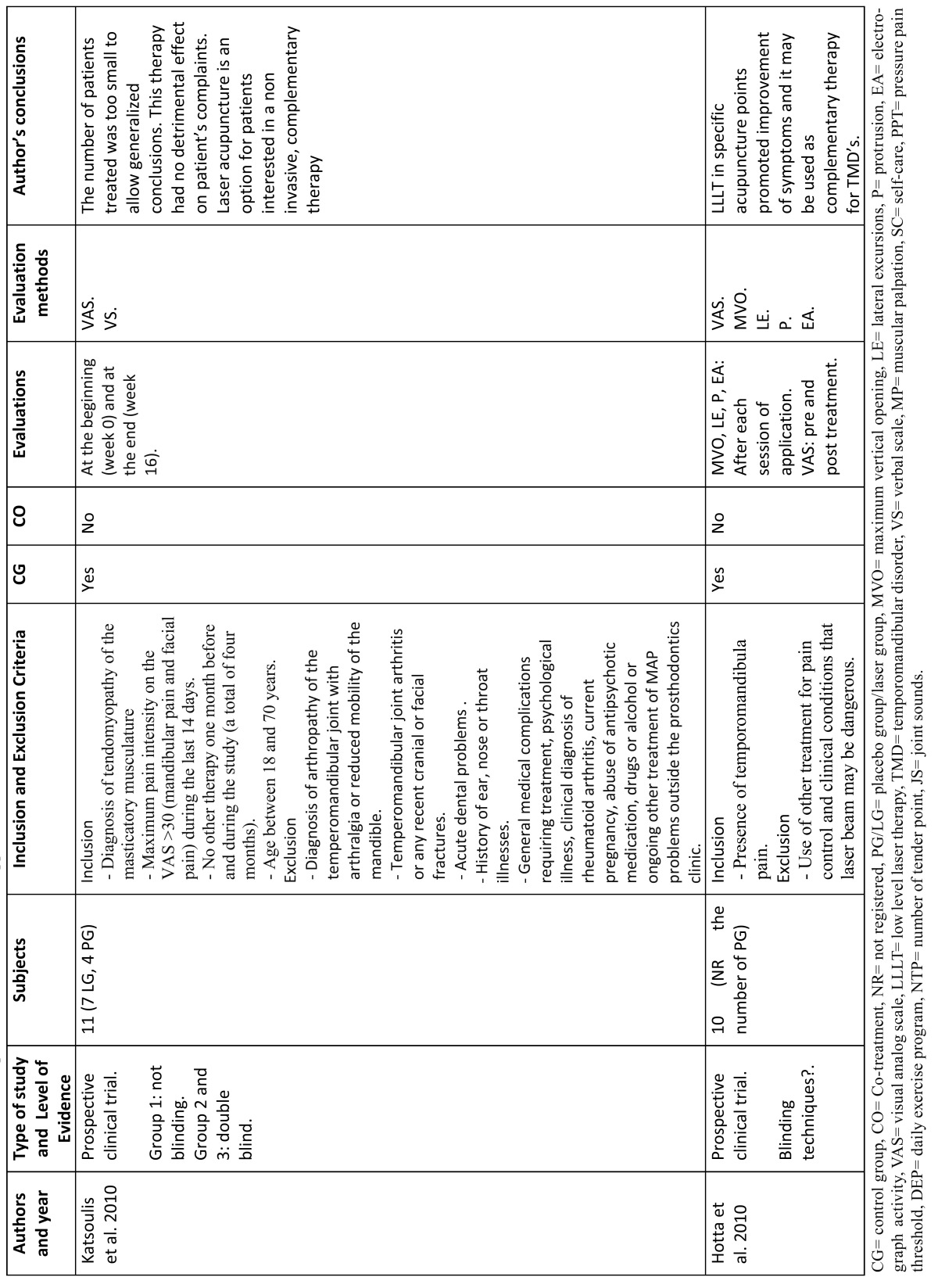
Clinical trials with acupunture and low level laser application.

**Table 4:**
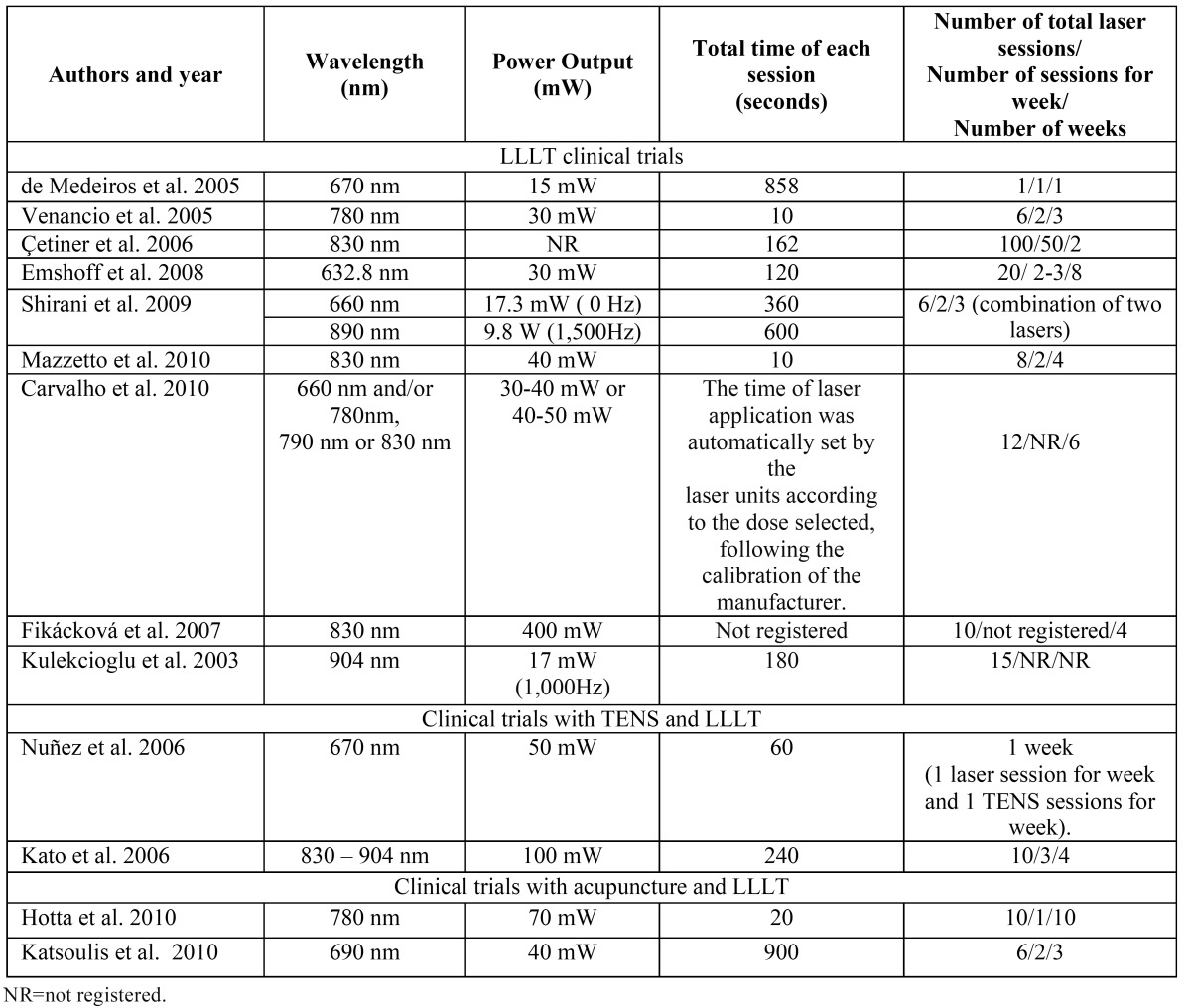
Low level laser technical characteristics.

## Discussion

Many clinical applications of laser light can be found in medicine, dentistry, surgery and many types of lasers in different wavelengths have been offered clinicians and researchers (9). The use of LLLT has gained much popularity in recent years as a method of management of many localized, painful, musculoskeletal conditions (9).

LLLT makes use of the electromagnetic radiation of a single wavelength, usually in the red or infrared regions. LLLT provides treatment for several pathologies, including impaired wound healing, pain conditions, and inflammatory situations (10).

Its basic effects are bio-stimulative, regenerative, analgesic and antinflammatory. It also seems to act on the immune, circulatory and haematological systems (3). The mechanism of analgesic effect of LLLT is not well understood, but according to some reports, LLLT may promote analgesic effects via several mechanisms (e.g. increases liberation of endogenous opiates, increases urinary excretion of glucocorticoids, improves local microcirculation, increases lymphatic flow thus reducing edema, decreases permeability of the nerve cell membrane, decreases release of algesic agents in pathological sites, increases ATP production, decrease tissue asphyxia and acceleration of wound healing) (3,5,8,11-13). Other authors such as Gam et al. (14), suggested that there is no scientific evidence to show that laser light can penetrate deeper structures, and some studies questioned the clinical an biological benefits of the physical therapy in the treatment of musculoskeletal pain, while other authors demonstrate the effectiveness of the low level laser therapy for musculoskeletal disorders (2,9).

The importance of investigating the actual analgesic efficacy of LLLT lies on the fact that TMD symptoms have been treated by a wide array of methods separately, such as interocclusal splint, medication, physical therapy, and transcutaneous electric nerve stimulation; in most cases, however, better outcome is achieved when the therapies are associated, where lasers can be of great value (12).

LLLT is a noninvasive, quick and safe, non-pharmaceutical intervention that may be beneficial for patients with TMJ pain disorders (4,11). Like in any therapy, patients respond similarly to LLLT. Patient response depend not only on the type of laser, but also on the target tissue an immunological system conditions. An unsatisfactory outcome can be due to very low or high dose, incorrect diagnosis, small number of sessions, inadequate energy density, among others (12).

Publications are scarce on the specific case of using LLLT on TMDs. Our research found only 35 which related the two terms.

The relative clinical efficacy of LLLT for treatment of TMD is controversial (4). For most authors, such as Kulekcioglu et al. (5) Fikácková et al. (7), Carvalho et al. (8), Çetiner et al. (9), Nuñez et al. (10), Shirani et al. (11), Mazzetto et al. (12), Medeiros et al. (13), Kato et al. (2) and Hotta et al. (15) demonstrated that LLLT is an effective therapy for the pain control in subjects with TMD, while other studies, like those published by De Abreu Venancio et al. (3), and Emshoff et al. (4), presented controversial results. Medlicott and Harris and McNeely et al. supported that the use of LLLT may improve the treatment results of TMD (16,17). Due to utilization of different parameters such as wavelength, power, irradiation time, beam area at the skin, energy/energy density, number of treatments and interval between treatments of laser radiation in various patients groups, the results could not have been standardized (4,18).

Light penetration and absorption in biological tissue are dependent on several variables, and one of the most important is the wavelength of the laser. Different wavelengths have been used for treatment of TMDs: 632.8 nm neon–helium (He–Ne) laser (4), 670 nm (10,13), 690 nm (19), 780 nm (3,15), 830 nm (2,7,9,12), 890 nm (11), wavelengths of 830 nm to 904 nm (2) and 904 nm (5) (2). Carvalho et al. (8) used a combination of different wavelengths: 660 (red laser) and/or 780 nm, 790 nm or 830 nm (infrared laser), thinking that the association of red and infrared laser light could be effective in pain reduction on TMD’s. The same results are were presented by Shirani et al. (11) who reported that the combination of two wavelength 660 nm (InGaAIP visible red light) and 890 nm (infrared laser), were proven to be effective treatments for pain reduction in patients with myofascial pain dysfunction syndrome.

Emshoff et al. (4) used a 632 nm rather than the more typical choices of 830 nm or 904 nm. They reported that a 632.8 nm wavelength penetrates more deeply into musculoskeletal tissues than shorter wavelengths. It was also reported a pain reduction with 632 nm compared to 820 nm. These results are in accordance with Brosseau et al. (20) who reported that here were no statistical difference between wavelengths. However, there was a trend for improved outcome with the 632nm compared to 820 nm for pain although the confidence limits overlap [SMD 632 nm: -0.7 (95% CI: -1.2, -0.3) vs SMD 820 nm: -0.4 (95% CI: -0.8, 0.1)].

Concerning the energy density in the different studies reviewed, it is possible to observe a great diversity, since that has still not been any definite consensus about. De Medeiros et al. (13) recommend an applied energy density of 2 J/cm2, Venancio et al. (3) 6•3 J/cm2, Emshoff et al. (4) 1,5 J/cm2,Fickácková et al. (7) 10 or 15 J/cm2, Carvalho et al. (8) 1-2 J/cm2, Çetiner et al. (9) 7 J/cm2, Shirani et al. (11) 6.2 J/cm2 and 1 J/cm2, Mazzeto et al. (12) 5 J/cm2, Kulekcioglu et al. (5) and Nuñez et al. (10) 3 J/cm2, Kato et al. (2) 4 J/cm2 and Hotta et al. (15) 35 J/cm2. The radiation penetration depth is also a controversial issue, and more objective data about tissue optics is necessary (10). Kulekcioglu et al. (5), suggested that infrared laser penetrates deeper than ultraviolet laser, and is most effective between the frequency ranges of 700- 1000Hz.

Further studies are required to establish a radiation time and energy dose for significant effects on pathological conditions (9). Given the large range of treatment parameters involved in this therapy (i.e. wavelength, fluence, intensity, exposure time, total duration of the treatment), it is not difficult to understand that results differ from one study to the next (10). Bjordal et al. (21) refers that literature on LLLT is full of conflicting reports, which is caused by the lack dosage consensus, suggesting that some poor results in some studies may have been caused by insufficient irradiation.

Kulekcioglu et al. (5) and Çetiner et al. (9), reported a reduction of pain and chewing difficulties in myogenic TMDs, referring that one month follow-up is a meaningful time to get effective results with LLLT.

Most of the reviewed studies evaluated the patients using a VAS (2-5,8,9,11,12,15,19) fact that makes very important to remark the psychological component. Patients with diagnoses of TMDs are rendered susceptible to placebo effects of any treatment carried out and has been shown to be effective in more than 40% of the cases (10). The conflicting results may be due too for the placebo effects in the treatment period (9), psychological factors, such as the desire to feel better, may have influenced physiological processes thereby resulting in the desired outcome (4). Venancio et al. (3) suggested that the power of the placebo effects has been widely demonstrated in the treatment of TMDs because a good relationship between professional and patient, associated with the appearance of the high technology of the laser, might explain the VAS reduction for laser and placebo groups in clinical control group trials. Kulekcioglu et al. (5) reported that pain was significantly improved in the placebo group and this might be explained in two ways; the placebo effect which is frequently encountered when evaluating subjective symptoms in similar studies and the indirect influence of daily exercise program. The literature has associated placebo analgesia with 2 potential mechanisms: one sustained and engaged for the duration of placebo analgesia, the other transitory, that is the feedback mechanism (22). In the others parameters, significantly improvements were found, only in the laser group. Double blind studies are more appropriate when a new therapeutic modality is being tested, because the placebo effect seems to be very strong, especially in chronic patients (3).

Other or additional way to evaluate the patients is by measuring the different jaw movements (3,5,9,10,12,15). On the other hand, de Medeiros et al. (13) studied the effect of 670 nm on the bite strength of the masseter muscle using a gnathodynamometer and observed and improvement in muscle contraction strength in all patients with only one application of 14 minutes. (13) They remark that the placebo effect did not affect the measurement of bite strength since it is evaluate before treatment, after placebo lamp session and after laser treatment. The use of this kind of devices, like the algometer, is an attempt to quantify pain better, standardizing data collection and making their comparison possible (3).

Hotta et al. (15) and Katsoulis et al. (19) studied the effect of LLLT in acupuncture points, and they concluded that laser acupuncture is a good complementary therapy option for patients with TMDs. Katsoulis et al. (19) reported that the effectiveness of LLLT seems to be comparable to that splint therapy; however it is less costly and less time consuming. On the other way, Kato et al. (2) and Nuñez et al. (10) compared LLLT with the TENS therapy and reported a stronger analgesic effect and greater improvement with LLLT than with TENS, but both therapies show good results for the treatment of TMDs.

Few clinical studies, systematic reviews and meta-analysis investigated the efficacy of the LLLT in other musculoskeletal disor-ders and pain relief. Chow et al. (23), in a systematic review, evaluated the efficacy of LLLT in the management of neck pain, and concluded that the LLLT reduces pain immediately after treatment in acute neck pain and up to 22 weeks after completion of treatment in patients with chronic neck pain. These results are consistent with a double blind, randomized, placebo- controlled study published by themselves (24). Bjordal et al. (21), in other systematic review, analysed the efficacy of LLLT in pain reduction associated in chronic joint disorders. They also concluded that LLLT, in correct doses, can reduce significantly the pain and improve health status in chronic joint disorders. Brosseau et al. (20) also made a systematic review about the efficacy of LLLT in the treatment of rheumatoid arthritis. It was concluded that LLLT could be considered in short-term treatment for pain relief and morning stiffness for rheumatoid arthritis patients, particularly since there were few side-effects. Brosseau et al. (20) Bjordal et al. (21), and Chow et al. (23), considered that the interpretation of the results should be taken with caution because there was heterogeneity in patient samples, treatment procedures and trial design, remarking the need of further investigations(20,21,23).

Jenkins and Carroll (18), in their report explain that there is no consensus among manufactures in the way they measure and present the specifications of their devices complicating even more this issue. Without some standardization the studies are not reproducible, and outcomes in clinical research and practice will not be consistent. These authors propose a standardized tabular format, in attempt to provide a standardized method for presenting what amount to a quite comprehensive set of parameters, and suggest accompanying procedures for this and other Journals to follow to ensure compliance by authors (18).

Publications on the use of LLLT for treatment of TMDs are limited. A problem detected in this literature reviewed is the variation in methodology, dosimetry and other parameters between studies, and the inclusion criteria and diagnosis of the patients. The studies are not standardized and consequently the results differ and comparison is difficult.

According to the principal of evidence-based dentistry, there is currently a scientific evidence level B in favor of using LLLT for treatment of TMDs. The results published in the literature should be analyzed with caution since none have sufficient scientific basis, either because the sample size is inadequate, or methodological defects are present.

We believe that the diagnosis based on the Research Diagnostic Criteria for TMD (RCD/TMD) proposed for Dworkin and LeReserche (6) and the use of tabular format proposed for Jenkins and Carroll(18), could standardize the clinical examination for the use of LLLT in patients with TMDs, improving reproducibility among clinicians, and facilitating comparison of results among researchers.

Furthermore controlled double- blind clinical trials and multicentric studies are necessary to demonstrate the efficacy of LLLT in TMDs.
